# Role of sand lizards in the ecology of Lyme and other tick-borne diseases in the Netherlands

**DOI:** 10.1186/1756-3305-3-42

**Published:** 2010-05-14

**Authors:** Ellen Tijsse-Klasen, Manoj Fonville, Johan HJ Reimerink, Annemarieke Spitzen - van der Sluijs, Hein Sprong

**Affiliations:** 1 Laboratory for Zoonoses and Environmental Microbiology, National Institute for Public Health and Environment (RIVM), Antonie van Leeuwenhoeklaan 9, PO Box 1, Bilthoven, the Netherlands; 2 Laboratory for Infectious Diseases and Screening, National Institute for Public Health and Environment (RIVM), Antonie van Leeuwenhoeklaan 9, PO Box 1, Bilthoven, the Netherlands; 3 Reptile, Amphibian and Fish Conservation Netherlands (RAVON), PO Box 1413, 6501 BK Nijmegen, the Netherlands

## Abstract

**Background:**

Lizards are considered zooprophylactic for almost all *Borrelia burgdorferi *species, and act as dilution hosts in parts of North America. Whether European lizards significantly reduce the ability of *B. burgdorferi *to maintain itself in enzootic cycles, and consequently decrease the infection rate of *Ixodes ricinus ticks *for *B. burgdorferi *and other tick-borne pathogens in Western Europe is not clear.

**Results:**

Ticks were collected from sand lizards, their habitat (heath) and from the adjacent forest. DNA of tick-borne pathogens was detected by PCR followed by reverse line blotting. Tick densities were measured at all four locations by blanket dragging. Nymphs and adult ticks collected from lizards had a significantly lower (1.4%) prevalence of *B. burgdorferi *sensu lato, compared to questing ticks in heath (24%) or forest (19%). The prevalence of *Rickettsia helvetica *was significantly higher in ticks from lizards (19%) than those from woodland (10%) whereas neither was significantly different from the prevalence in ticks from heather (15%). The prevalence of *Anaplasma *and *Ehrlichia *spp in heather (12%) and forest (14%) were comparable, but significantly lower in ticks from sand lizards (5.4%). The prevalence of *Babesia *spp in ticks varied between 0 and 5.3%. Tick load of lizards ranged from 1 - 16. Tick densities were ~ 5-fold lower in the heather areas than in woodlands at all four sites.

**Conclusions:**

Despite their apparent low reservoir competence, the presence of sand lizards had insignificant impact on the *B. burgdorferi *s.l. infection rate of questing ticks. In contrast, sand lizards might act as reservoir hosts for *R. helvetica*. Remarkably, the public health risk from tick-borne diseases is approximately five times lower in heather than in woodland, due to the low tick densities in heather.

## Background

Several tick-borne diseases are emerging in Europe as shown by the example of a dramatic increase of Lyme disease cases over the last decade [1,2]. Furthermore, an increasing prevalence and number of *Rickettsia *species have been identified as causative agents of tick-borne rickettsioses in Europe [3,4]. The underlying causes for the spread of zoonotic diseases such as these include human behaviour, mankind's irrepressible impact on the environment and a changing climate [5-8]. They are affecting the highly dynamic and only partially understood interactions between infectious agents, their hosts, vectors and the environment. The predominant vector of *B. burgdorferi *in Europe is *Ixodes ricinus*. This tick species feeds on a wide variety of warm- and cold-blooded vertebrates, including humans. Some of these tick hosts are incompetent hosts for various *B. burgdorferi *genospecies. The hypothesized origin of such specific associations is that different groups of vertebrates exhibit distinct types of innate immunity, which may either tolerate or destroy certain *B. burgdorferi *genospecies [9-11]. Incompetent hosts, in contrast to competent ones, fail to acquire, maintain or transmit a parasitic microorganism and therefore do not contribute to or even limit the spread of tick borne pathogens.

At least eight lizard species, including *Lacerta agilis *(sand lizards), have been identified as incompetent hosts for all *B. burgdorferi *genospecies, except *B. burgdorferi lusitaniae *[12-21]. The blood of some lizard species was found to possess borreliacidal properties and therefore these lizards are even able to clear the bacteria from previously infected ticks [22-24]. The presence of such an incompetent host might reduce the ability of *B. burgdorferi *to maintain itself in an enzootic cycle [25,26] and might act zooprophylactic by reducing the prevalence of *B. burgdorferi *in ticks and diverting tick bites away from competent hosts [15,27]. This dilution effect will only apply if the vector has multiple hosts and the incompetent host is an important feeding source for the vector [26,28]. Lizards have been shown to contribute to the dilution effect in several areas in the United States of America [29,30]. In the Netherlands lizards occur on heath, the coastal dunes and in open woodlands on the higher sandy soils in the eastern, southern and central part of the country. In some of these areas lizard populations are dense with up to 100 individuals per hectare [31,32].

Various studies in the Netherlands and other European countries evaluated the prevalence of *I. ricinus *on sand lizards. The mean number of ticks per lizard varied from 0.2 to 23 and maximal numbers of ranged from 42 to 61 in different study areas [33-35]. These studies indicate that in certain habitats lizards are relevant hosts for *I. ricinus*. Small rodents, probably the most relevant *B. burgdorferi *reservoir in the Netherlands, serve mainly as hosts for the larval stage of *I. ricinus *while sand lizards feed often a much higher proportion of nymphs (larvae/nymph ratio 1.6 compared to 14 - 39 for small rodents) [16,36]. Due to the low larvae/nymph ratio on sand lizards their zooprophylactic effect should theoretically be high. Previous studies suggest that sand lizards are incompetent hosts for most *B. burgdorferi s.l species *except *B. burgdorferi lusitaniae *[15,20], but their effects on *B. burgdorferi *prevalence in questing ticks have not been investigated.

Recently, *Rickettsia helvetica *was identified the most prevalent potentially pathogenic *Rickettsia *in Dutch ticks [37,38]. As *R. helvetica *can be transmitted vertically from one tick generation to the next by trans-stadial and transovarial transmission it is less dependent on the presence of competent vertebrate hosts and therefore a dilution effect is less likely to occur [37,39]. Nevertheless, large variations in infection rates and an increased prevalence in adult ticks have been observed and indicate that vertebrate hosts contribute to the maintenance of *Rickettsia *in tick populations [37,40]. A few vertebrates have been identified as competent hosts for *R. helvetica *whereas no incompetent hosts have been found so far [37,41]. To date, the pathogenic potential of *R. helvetica *is unclear, but infection with *R. helvetica *has been suspected in acute perimyocarditis, unexplained febrile illness and sarcoidosis [42-49].

*Ehrlichia *and *Anaplasma *species are intracellular bacteria that can be transmitted by tick bites. The causative agent for human monocytic anaplasmosis is *Ehrlichia chaffeensis *[50] and an other medically important member of this group is the human granulocytic anaplasmosis agent (HGA), *Anaplasma phagocytophilum *[51]. A recent study on *Anaplasma *and *Ehrlichia *in Dutch ticks found mainly *Ehrlichia schotti *variant, which is of unknown pathogenicity [52].

Various *Babesia *species are known to cause disease in humans, cattle and companion animals and are usually transmitted by tick bites. In Europe, *Babesia divergens *is thought to be the most important species to cause human disease but other species have recently been identified as human pathogens in Europe as well [53]. The protozoa invade red blood cells and can cause severe disease. The increase of immuno-compromised and splenectomized individuals in modern society has also led to an increase in the number and intensity of diagnosed babesioses [53,54]. *B. microti *and a *Babesia *EU1 (proposed name *Babesia venatorum*) have been repeatedly been identified in ticks in the Netherlands [55].

Here, we investigated the role of sand lizards in the ecology of tick-borne pathogens, in particular *B. burgdorferi *s.l. and *R. helvetica*. A non-invasive field study was set up which includes the collection of ticks from lizards and vegetation in their habitats as well as from vegetation in geographically nearby control areas that did not serve as a lizard habitat. Tick and lizard densities as well as the infection rate of the different habitats were determined and compared.

## Materials and methods

### Collection of ticks and lizards

Field sampling was conducted between May and October in the years 2007 till 2009. Ticks were collected by flagging vegetation as described by Wielinga *et al*. in four different geographical upcountry areas (Heumensoord, Bergherbos, Leusderheide, Hullenberg) from both heathland (sand lizard habitat) and forest areas (unsuitable lizard habitat), which were directly adjacent to each other [52]. Sand lizards do not occur in dense forest therefore this type of landscape was categorized as unsuitable habitat whereas heathland, their main habitat, was categorized as suitable. No lizards were encountered during the flagging in the forest areas. Lizards were captured by hand and ticks attached to the lizards were counted and collected. Sex of the lizard was determined on site and the location of the attached ticks noted. The lizards were returned to their site of capture immediately after examination and tick removal. Ticks were immersed in 70% ethanol and stored at -20°C upon arrival in the laboratory. Based on morphological criteria tick species and stage were determined.

### Measuring tick and lizard densities

Tick densities were estimated at the different sampling locations by dragging a 100 cm wide flannel cloth over a distance of 100 m with checking for ticks every 25 m. Nymphs and adult ticks were collected from the cloth and counted. This was repeated four times at each location and the counts were averaged. Tick densities of adjacent heath and forest areas were determined on the same day and under equal weather conditions. Lizard densities were estimated by expert judgment, based on the yearly monitoring data from all study areas [56].

### Nucleic acid extraction, polymerase chain reactions and reverse line blotting

Total DNA was extracted from the ticks by alkaline lysis as described elsewhere [57]. DNA extracts were stored at -20°C. The presence of the DNA of different tick-borne pathogenic species (*Rickettsia *spp., *B*. *burgdorferi *s.l., *Ehrlichia/Anaplasma *spp. and *Babesia *spp.) was determined by polymerase chain reaction (PCR) followed by reverse line blotting (RLB) as described before [52,57,58]. The probes that were used for RLB analysis of the PCR products can be found in Table 1.

**Table 1:** Primers and probes used in this study for PCR and RLB. Reverse primers were labeled with Biotin-tetraethyleneglycol. All probes were 5'-amino-labeled.

Name	Sequence (5' - 3')	Type	Target	Species	Reference
5S borSeq	GAGTTCGCGGGAGAGTAGGTTATTGCC^(1)^	Primer	23S-5S IGS	*B. burgdorferi *sensu lato	[52]
23S borSeq	TCAGGGTACTTAGATGGTTCACTTCC	Primer	23S-5S IGS	*B. burgdorferi *sensu lato	[52]
A-borsl1	CTTTGACCATATTTTTATCTTCCA	Probe	23S-5S IGS	*B. burgdorferi *sensu lato	[68]
A-borsl2	CTTCCATCTCTATTTAGCCAATTT	Probe	23S-5S IGS	*B. burgdorferi *sensu lato	[52]
A-borsl3	TATTTTTATCTTCCATCTCTATTTT	Probe	23S-5S IGS	*B. burgdorferi *sensu lato	[52]
B31-A-s. stricto	AACACCAATATTTAAAAAACATAA	Probe	23S-5S IGS	*B. burgdorferi *sensu stricto	[68]
Ga2-garinii	AACATGAACATCTAAAAACATAAA	Probe	23S-5S IGS	*B. garinii*	[68]
Vs46lN2afzelii	AACATTTAAAAAATAAATTCAAGG	Probe	23S-5S IGS	*B. afzelii*	[68]
VsII62 val	CATTAAAAAAATATAAAAAATAAATTTAAGG	Probe	23S-5S IGS	*B. valaisiana*	[68]
A-Ruski	GAATAAAACATTCAAATAATATAAAC	Probe	23S-5S IGS	*B. afzelii (variant ruski)*	[69]
A-LusiP	CAAAAAAATGAACATTTAAAAAC	Probe	23S-5S IGS	*B. lusitaniae*	[58]

B-GA1b	CGGGATCCCGAGTTTGCCGGGACTTCTTCT^(1)^	Primer	*16SrRNA*	*Ehrlichia*/*Anaplasma*	[57]
16S8Fe	GGAATTCAGAGTTGGATCMTGGYTCAG	Primer	*16SrRNA*	*Eubacteria*	[70]
Ehr-all	TTATCGCTATTAGATGAGCC	Probe	*16SrRNA*	*Anaplasma *genus	[57]
A-HGE	GCTATAAAGAATAGTTAGTGG	Probe	*16SrRNA*	HGE agent	[57]
A-Eqph	TTGCTATAAAGAATAATTAGTGG	Probe	*16SrRNA*	*A. phagocytophilum*	[57]
A-dHGE	GCTATGAAGAATAGTTAGTG	Probe	*16SrRNA*	HGE agent (variant)	[57]
A-dPh	TTGCTATGAAGAATAATTAGT	Probe	*16SrRNA*	*A. phagocytophilum variant*	[52]
A-E. Schot	GCTGTAGTTTACTATGGGTA	Probe	*16SrRNA*	*E. schotti *(variant)	[57]
A-murisT	AGCTATAGGTTTGCTATTAGT	Probe	*16SrRNA*	*E. muris *T variant	[69]
A-Chaff	ACCTTTTGGTTATAAATAATTGTTA	Probe	*16SrRNA*	*E. chaffeensis*	[57]
A-can	TCTGGCTATAGGAAATTGTTA	Probe	*16SrRNA*	*E. canis*	[57]
A-Wolbach	CTACCAAGGCAATGATCTA	Probe	*16SrRNA*	*Wolbachia*	[52]

Rick-16S rev	ACTCACTCGGTATTGCTGGA^(1)^	Primer	*16SrRNA*	*Rickettsia *genus	[58]
Rick-16S for	AACGCTATCGGTATGCTTAACA	Primer	*16SrRNA*	*Rickettsia *genus	[58]
A-Rickall	TTTAGAAATAAAAGCTAATACCG	Probe	*16SrRNA*	*Rickettsia *genus	[58]
A-Rhelv2	GCTAATACCATATATTCTCTATG	Probe	*16SrRNA*	*R. helvetica*	[58]
A-Rconor	CTTGCTCCAGTTAGTTAGT	Probe	*16SrRNA*	*R. conorii*	[58]
A-16SRickIRS	GTATATTCTCTACGGAAAAAA	Probe	*16SrRNA*	*Rickettsia *IRS3	[58]
A-RProwaz	CGGATTAACTAGAGCTCGCT	Probe	*16SrRNA*	*Rickettsia prowazekii*	This study
A-RTyphi	CGGATTAATTAGAGCTTGCT	Probe	*16SrRNA*	*Rickettsia typhi*	This study
A-NonHelv A	AATACCGTATATTCTCTACGGA	Probe	*16SrRNA*	Non-*Rickettsia helvetica*	This study
A-NonHelv B	AATACCGTATATTCTCTGCGGA	Probe	*16SrRNA*	Non-*Rickettsia helvetica*	This study

BATH-Rn	TAAGAATTTCACCTCTGACAGTTA^(1)^	Primer	*18SrRNA*	*Babesia *genus	[55]
BATH-Fn	ACACAGGGAGGTAGTGACAAG	Primer	*18SrRNA*	*Babesia *genus	[55]
Catch all 2	GTAATGGTTAATAGGARCRGTT	Probe	*18SrRNA*	*Babesia *genus	[55]
Ba-div	GTTAATATTGACTAATGTCGAG	Probe	*18SrRNA*	*B. divergens*	[71]
Ba-mic 1	CCGAACGTTATTTTATTGATTT	Probe	*18SrRNA*	*B. microti*	This study
Ba-mot	GCTTGCTTTTTTGTTACTTTG	Probe	*18SrRNA*	*B. motasi*	[55]
Ba-mic 2	GRCTTGGCATCWTCTGGA	Probe	*18SrRNA*	*B. microti*	[55]
Ba-EU1	CTGCGTTATCGAGTTATTG	Probe	*18SrRNA*	*B. EU1*	This study

^(1) ^Reverse primer 5'-labeled with biotine tetraethyleneglycol

### B. burgdorferi s. l

The 23S-5S intergenic spacer region of *B. burgdorferi *s. l. was amplified by PCR with the HotStarTaq master mix (Qiagen, Venlo, The Netherlands) with the following conditions: 15 min 94°C, then cycles of 20 s 94°C, 30 s 70°C, 30 s 72°C lowering the annealing temperature 1°C each cycle till reaching 60°C, then 40 cycles at this annealing temperature and ending by 7 min 72°C.

### Rickettsia spp

The 16S rRNA gene of *Rickettsia *species was amplified by PCR with the HotStarTaq master mix with the following conditions: 15 min 94°C, then cycles of 20 s 94°C, 30 s 72°C, 30 s 72°C lowering the annealing temperature 1°C each cycle till reaching 62°C, then 40 cycles at this annealing temperature followed by a final elongation step for 7 min at 72°C. For this study we designed two new RLB probes that are able to hybridize to DNA of most *Rickettsia *species except for *R. helvetica *and closely related species. These probes were designed with the purpose to establish the occurrences of possible multiple infections of ticks with *Rickettsia *species. Newly designed probes were tested with a series of samples and positive controls before incorporating them in the assay (not shown). In a second PCR (forward primer: AGAGTTTGATCCTGGCTCAGAAC, reverse primer: CCTACGGCTACCTTGTTACGACTT) on a small subset of samples a longer fragment of the 16S rRNA gene was amplified and sequenced to be able to compare the sequences in more detail.

### Other tick-borne pathogens

The 16S rRNA gene of *Ehrlichia *and *Anaplasma *species was amplified by PCR with the HotStarTaq master mix with the following conditions: 15 min 94°C, then cycles of 20 s 94°C, 30 s 65°C, 30 s 72°C lowering the annealing temperature 1°C each cycle till reaching 55°C, then 20 cycles at this annealing temperature and an additional 20 cycles with an annealing temperature of 63°C followed by a final elongation step for 7 min at 72°C.

The 18S rRNA gene of *Babesia *was amplified by PCR using a reaction mix containing 1× buffer, 1.75 mM magnesium chloride, 200 μM dNTPs, 0.25 μM of each primer and Taq polymerase. The PCR reaction was run under following conditions: 10 min 94°C, then cycles of 20 s 94°C, 30 s 67°C, 30 s 72°C lowering the annealing temperature 1°C each cycle till reaching 57°C, then 40 cycles at this annealing temperature followed by a final elongation step for 7 min at 72°C.

To minimize cross contamination and false-positive results, positive and negative controls were included in each batch tested by the PCR and RLB assays. Furthermore DNA extraction, PCR mix preparation, sample addition, and PCR analysis were performed in assigned separate labs. PCR products of some samples were sequenced by dideoxy-dye termination sequencing of both strands, and compared with sequences in Genbank http://www.ncbi.nlm.nih.gov/ using BLAST. The sequences were aligned and analyzed using BioNumerics 5.1 (Applied Maths, Kortrijk, Belgium).

### Statistical analysis

Data were analyzed using tools provided by OpenEpi [59] and Quantitative Parasitology 3.0 (QP3.0) [60]. Confidence intervals (95%) and two-tailed p-values were calculated using Fisher's exact test.

## Results

In total 713 ticks were collected: 491 from vegetation and 222 from lizards (Table 2). Ticks collected from lizards were mainly larvae and nymphs with larvae/nymph ratios varying from 0.17 to 2.7 (Table 3). As larvae were usually omitted during flagging, ticks collected from vegetation were mainly nymphs and only a few adults. Large variations in tick densities (nymphs+ adults) between the locations and between the two vegetation types were observed (Table 4). The number of questing nymphs and adults was significantly lower in heather vegetation than in adjacent forest (bootstrap analysis with 5000 repetitions conjointly for 4 locations: p < 0.0001). Ninety-two individual sand lizards (51 males, 39 females and 2 sub-adults) were captured in the study areas which had a total of 290 attached ticks. The number of ticks on individual lizards ranged from 1 - 16 individuals (Table 5), with an average of 3.3 (CI: 2.7 - 4.0) ticks per lizard. The tick load of males was not significantly different from females (bootstrap t-test with 2000 replications; p = 0.6). Almost all ticks (265 of 290) were found in the armpits of the forelimbs (Table 6), which was in agreement witch earlier studies [33].

**Table 2:** Numbers of ticks collected from lizards and vegetation in the four study areas which were tested positive for tick-borne pathogens by PCR-RLB.

Location	Heumensoord			Bergherbos			Leusderheide			Hullenberg		
**RLB positive ticks** **number (%)**	**Lizards** **(n = 48)**	**Heather** **(n = 39)**	**Forest** **(n = 50)**	**Lizards** **(n = 61)**	**Heather** **(n = 40)**	**Forest** **(n = 41)**	**Lizards** **(n = 55)**	**Heather** **(n = 70)**	**Forest** **(n = 76)**	**Lizards** **(n = 58)**	**Heather** **(n = 57)**	**Forest** **(n = 118)**

*B. burgdorferi*	0 (0)	3 (8)	5 (10)	0 (0)	7 (18)	4 (10)	0 (0)	14 (20)	20 (26)	3 (5)	25 (44)	25 (21)

*B. b. sensu stricto*	0	1	1	0	0	1	0	1	0	1	0	1

*B. garinii*	0	0	2	0	0	0	0	0	2	0	0	1

*B. afzelii*	0	2	2	0	7	2	0	13	18	2	25	23

*B. valaisiana*	0	0	0	0	0	1	0	0	1	0	0	0

Ehrlichia/Anaplasma	0 (0)	0 (0)	1 (2)	11 (18)	4 (10)	3 (7)	1 (2)	6 (9)	16 (21)	0 (0)	15 (26)	20 (17)

*E. schotti*	0	0	0	10	4	3	1	6	14	0	13	17

*A. phagocytophilum*	0	0	0	0	0	0	0	0	0	0	0	2

Unspeciated	0	0	1	1	0	0	0	0	2	0	2	1

Rickettsia	16 (33)	11 (28)	13 (26)	9 (15)	3 (8)	2 (5)	7 (13)	9 (13)	6 (8)	10 (17)	10 (18)	7 (5,9)

*R. helvetica*	16	7	12	8	1	2	1	7	5	9	10	5

*R. typhi*	0	0	0	0	1	0	2	0	0	0	0	0

Unspeciated	0	4	1	1	1	0	4	2	1	1	0	2

Babesia	0 (0)	0 (0)	0 (0)	3 (5)	0 (0)	1 (2)	0 (0)	0 (0)	0 (0)	2 (3,4)	3 (5,3)	5 (4,2)

*B. microti*	0	0	0	2	0	0	0	0	0	1	3	3

*B. EU1*	0	0	0	1	0	0	0	0	0	1	0	2

Unspeciated	0	0	0	0	0	1	0	0	0	0	0	0

Percentage of positives are in brackets.

**Table 3:** Tick stages collected from vegetation and lizards.

	Heumensoord			Bergherbos			Leusderheide			Hullenberg		
	Lizards	Heather	Forest	Lizards	Heather	Forest	Lizards	Heather	Forest	Lizards	Heather	Forest
L	21	0	0	8	2	2	38	17	0	11	0	0

N	21	31	46	48	25	34	14	46	76	46	56	112

F	1	7	1	0	2	2	0	1	0	0	0	0

M	0	1	3	0	11	0	0	1	0	0	0	5

U	5	0	0	5	0	3	3	5	0	1	1	1

L/N	1.0	n/a	n/a	0.2	n/a	n/a	2.7	n/a	n/a	0.2	n/a	n/a

Identified with aid of a light microscope.

L: larvae; N: nymph; F: female; M: male; U: unidentified; L/N ratio: larvae/nymph ratio; n/a: not applicable

**Table 4:** Density of ticks in heather and forest.

Location	Heumensoord		Bergherbos		Leusderheide		Hullenberg	
	Heather	Forest	Heather	Forest	Heather	Forest	Heather	Forest
Tick density [100 m^-2^]	12.5	72.5	3.3	16.5	19.5	102	8.5	51.0

95% CI	10-15	65-79	1-6	11-22	15-24	90-110	5-12	42-61

Lizard density [ha^-1^]	< 50	0	75	0	100	0	100	0

Ticks per lizard	2.7 (18)		2.4 (25)		2.9 (19)		1.9 (30)	

Values are averages of 4 measurements and a 95% confidence interval was calculated with bootstrap analysis with 5000 replications (QP 3.0). Lizard densities were estimated based on data of the national reptile monitoring network [56].

**Table 5:** Number of ticks calculated per sand-lizard, per age-, and sex group.

	n	range ticks/lizard	mean ticks/lizard	95% CI
Male	51	1 - 11	3.2	2.5 - 4.1

Female	39	0 - 16	3.6	2.7 - 4.8

Sub-adult	2	1 - 2	1.5	n.c.

Total	92	0 - 16	3.3	2.8 - 4.0

n.c.: not calculated.

Data are from all four study areas. Mean and confidence intervals were calculated with bootstrap analysis with 5000 replications (QP 3.0) [60].

**Table 6:** Attachment sites of ticks.

	left	right	total
Flanks	9	13	22

Armpit	120	145	265

near eye	1	0	1

In/near tympanum	1	1	2

The number of ticks counted at the indicated sites is shown, all data is pooled for the four study sites.

### B. burgdorferi s.l

PCR and RLB showed that *B. burgdorferi *s.l., *Rickettsia *and *Ehrlichia/Anaplasma *species were present in ticks collected from all geographical areas (Table 2). The overall infection rate of all collected ticks was 15 percent (CI: 12% - 18%) for *B. burgdorferi *s.l. Notably, none of the ticks collected from lizards from Heumensoord, Bergherbos and Leusderheide were positive for *B*. *burgdorferi *s.l. while infection rates of ticks collected from vegetation in these areas varied from 8% (Heumensoord, heather; CI: 1.6% - 21%) to 26% (Leusderheide, woodland; CI: 17% - 38%). From the 58 ticks collected from lizards from the Hullenberg area, only three were positive for *B. burgdorferi *genospecies (one *B. burgdorferi sensu stricto*, two *B. burgdorferi afzelii*) while ticks from forest (woodland) and heather in this area had significantly higher infection rates of 21% (CI: 14% - 30%; p = 0.008) and 44% (CI: 31% - 58%; p = 0.000001), respectively. The only B. burgdorferi species associated with lizards, *B. lusitaniae*, was not detected in any tick tested during this study. The infection rates were not significantly different between questing ticks in forest (woodland) and heathers areas. The genospecies of *B. burgdorferi *isolates were determined by RLB. *B. afzelii *was the most prevalent *B. burgdorferi *species in ticks from all areas and vegetation types. Other species identified were *B. burgdorferi garinii *in a total of 5 ticks, all collected from woodlands, *B. burgdorferi *sensu stricto (n = 6) and *B. burgdorferi valaisiana *(n = 2, both from woodland). Eight samples hybridized with the catch-all probe for *B*. *burgdorferi *s.l. but could not be identified to the species level by RLB. The 5S-23S rRNA intergenic spacer regions of these samples were later sequenced and identified as *B. afzelii *(not shown).

### Rickettsia spp

**Figure 1:**
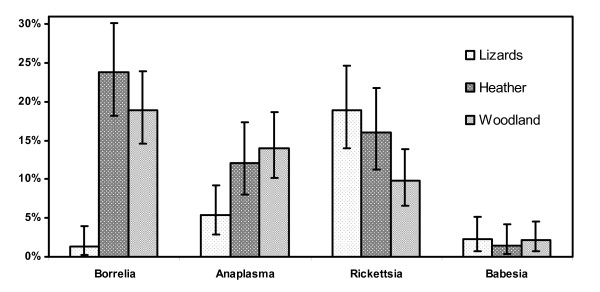
**Infection rates for *B. burgdorferi burgdorferi *s.l., Anaplasma spp., Rickettsia spp. and Babesia spp. of ticks collected from lizards, heather or woodland vegetation**. Four sampled areas were analyzed conjointly. Confidence intervals were calculated with Fisher's exact test [59]

*Rickettsi*a spp. were found in all four investigated areas in ticks collected from heather, woodland and lizards with high spatial variations and infection rates ranging from 5-33% (Table 2). Comparing forest (woodland) areas with heather areas there is an apparent but not statistically significant trend that ticks from heather have higher infection rates (p = 0.06) (Figure 1). Furthermore, the infection rates of ticks collected from lizards were significantly higher (p = 0.03) when the data of all locations were analyzed conjointly (Figure 1). Eighty-one percent of the detected *Rickettsia *spp. were identified as *R. helvetica *by RLB. The remaining positive samples hybridized with a *Rickettsia typhi *probe (n = 3) or the catch-all probe for *Rickettsia *spp. only. The 16S rRNA sequences of these samples were later identified as *Rickettsia bellii*-like sequences with 1 to 6 of about 356 nucleotides difference to *R. bellii *(Genbank accession No U11014). No infections with more than one *Rickettsia *species were found.

### Other tick-borne pathogens

*Ehrlichia/Anaplasma *spp. were found with infection rates between 0 and 26% with highly variable infection rates between the three areas and within the different tick groups. From all ticks collected from Heumensoord only one (0.8%) was positive for *Ehrlichia/Anaplasma *while in Hullenberg 15% were positive. All but nine positive ticks were identified as *Ehrlichia schotti*. Two were identified by RLB as *Anaplasma phagocytophilum*. The remaining seven samples could not be determined to the species level. Ehrlichia/Anaplasma prevalence was significantly higher in ticks collected from forest (p = 0.002) and heather (p = 0.02) than in ticks collected from lizards (Figure 1). The prevalence of ticks from forest and heather was not significantly different (p = 0.6). No *Babesia *spp. were detected in ticks from Leusderheide or Heumensoord. In Hullenberg a total of 10 ticks were positive while in Bergherbos three ticks sampled from lizards and one from the forest area were positive (Table 2).

## Discussion

Previous reports from North America and Europe indicated that lizards are incompetent hosts for most *B. burgdorferi *species and could therefore lead to dilution effects within tick populations [16,21,61]. In these studies, however, the *B. burgdorferi *prevalence in the questing tick population was often not addressed and control areas without lizards were not included [62]. The impact of lizards on other tick-borne pathogens had not been addressed either. Here, we included ticks from different heather and control areas and by tested for various pathogens. Heather with the same characteristics as the study area but without lizards would have been the most ideal control area, but unfortunately such areas were not available for this study. Therefore, nearby areas were chosen that did not suit as lizard habitats but also differed in vegetation type.

The current study confirms earlier observations ticks taken from lizards, including sand lizards, are almost free of *B. burgdorferi *s.l. [16,20,21,29,63]. *B. burgdorferi lusitaniae *which can be sustained in lizards, was not detected in this study [15]. Only very recently and in an unrelated study, we discovered *B. burgdorferi lusitaniae *in questing ticks in the Netherlands for the first time (not shown). This study indicates that sand lizards are probably incompetent hosts for *B. burgdorferi *species commonly present. To confirm the incompetence of sand lizards to acquire or transmit *B. burgdorferi *further studies and direct sampling of lizard tissue will be necessary.

We did not find evidence of a dilution effect of sand lizards since the *B. burgdorferi *prevalence in questing ticks collected from forest and heather vegetation were similar. How high the *B. burgdorferi *prevalence would be in the same habitat without lizards is unknown. Our data corroborate the previous notion that an incompetent host does not automatically act as a dilution host [26,28]. Studies on the effects of small rodent densities in combination with lizard densities (including the more abundant common lizard) in relation to *B. burgdorferi *infestation rate in questing and attached ticks would further deepen the knowledge on the specific characteristics of lizards as possible dilution hosts for *B. burgdorferi*. A high passive mobility of ticks would also lead to an attenuation of a dilution effect [64] that would be relatively high in small lizard habitats as studied here. An increased abundance of sand lizards might have effects on *B. burgdorferi *s.l., like a decrease of its overall prevalence in questing ticks, but also on the emergence of adapted species like *B. burgdorferi lusitaniae *and other tick-borne pathogens.

As found previously, *R. helvetica *was the most prevalent Rickettsia species found in The Netherlands [37]. *R. helvetica *is of uncertain pathogenicity but in recent years increasing numbers of publications confirm the pathogenic status of *R. helvetica *[42-49]. Scant evidence of *R. typhi *was found in three ticks. The DNA of *R. typhi *has been detected in tick lysates before, but its relevance is unclear [37]. Several isolates could not be identified to species level because their sequences did not match with sequences currently available at the NCBI database. Their closest matches were *R. bellii*.

High spatial variations of Rickettsia infection rates as found during this study had been observed in earlier studies at our laboratory and have also been reported by other groups [40]. Variations in Rickettsia prevalence at sites that are distant from each other can be due to a number of factors. Most rickettsial species can be transmitted transovarially and therefore their prevalence in a given tick population depends on the infection rate in the parent generation, the transovarial transmission rate and the presence of vertebrate reservoir hosts that can transmit the bacteria horizontally to previously uninfected ticks [65]. Rickettsia can therefore be endemic to a tick population without the obligatory need of reservoir hosts. In agreement with this, we previously reported similar *R. helvetica *prevalences in larvae, nymphs and adult ticks during a longitudinal study and concluded that ticks are a major reservoir for *R. helvetica *in the Netherlands [37]. Contrary Silaghi *et al*. reported much lower infection rates in nymphs than in adult ticks which might indicate the necessity of reservoir hosts to sustain rickettsial infection of a tick population on a long term [40]. Probably the maintenance of an infected population relies on both transmission pathways. The significantly higher prevalence of Rickettsia in ticks collected from sand lizards than those of questing ticks (forest and heather combined) is an indication that this host species might be a reservoir host for *R. helvetica*. Blood and tissue samples of lizards could have delivered additional information on the ability of sand lizards to maintain and transmit rickettsial infection but obtaining these would have been invasive and was therefore not possible.

For Anaplasma species the opposite picture was observed than for Rickettsia. Prevalence of Anaplasma was significantly lower in ticks collected from lizards compared to those from forest and heather vegetation indicating that lizards might be incompetent hosts for these pathogens.

A dilution effect for Anaplasma was not observed in the ticks collected from heather. In order to assess health risks for humans we attempted to calculate the infected-tick-density. The density of infected ticks is an approximation of the exposure risk and is therefore of importance when evaluating infection risks. Tick densities and density of infected ticks in forests were much higher (p < 0.0001) than in the corresponding heather areas the risk of exposure to a tick bite is higher as well. So even though the presence of lizards in heathland did not lead to a dilution effect, the risk of contracting Lyme disease is much lower in heathlands. The same is true for *R. helvetica*. Low tick density in heather had been reported previously [66,67] and can at least partly be explained by the typically sandy, free-draining grounds that provide little protection for the ticks against desiccation.

The four areas were classified as having very high (Leusderheide and Hullenberg), high (Bergherbos) and medium (Heumensoord) lizard densities. Surprisingly, the lizard density seems to be positively associated with *B. burgdorferi *prevalence in ticks. However, as lizard density data are only rough estimates, the positive association could not be tested with statistical methods. This study has shown that a single vertebrate species correlates with different tick-borne pathogens in opposing directions. While sand lizards are probably incompetent hosts for the *B. burgdorferi *species present in the Netherlands they seem to be reservoir hosts for *Rickettsia *spp. The different impacts of any single vertebrate host on tick-borne diseases will make it difficult to reduce the overall tick-borne disease risks for man and pets by manipulating vertebrate host communities. Reduction of tick-densities is a more immediate measure to reduce exposure and would evenly decrease the risk of all tick-borne diseases. A reduction of tick densities achieved by altering vegetation or reducing the abundance of vertebrate hosts for adult ticks is unpredictable and not ecologically acceptable. The low tick numbers in heather mean a decreased risk for Lyme disease or rickettsioses and with regard to tick-borne diseases heathland therefore seems a safer recreational area than woodland.

## Competing interests

The authors declare that they have no competing interests.

## Authors' contributions

ET and MF collected data, performed lab tests and developed new methodology. ET analyzed data and wrote initial draft. HS and JR designed the study and wrote the final manuscript. AS designed the study, collected data, provided data on lizard density, and co-refined the intellectual content of the manuscript.
